# Ethical and Methodological Challenges in Research With Hard-to-Reach Groups: Examples From Research on Family Caregivers for Migrant Older Adults Living With Dementia

**DOI:** 10.1093/geront/gnab179

**Published:** 2021-12-07

**Authors:** Hürrem Tezcan-Güntekin, Ilknur Özer-Erdogdu, Yüce Yilmaz-Aslan, Tugba Aksakal, Rona Bird

**Affiliations:** Department of Health and Education, Alice Salomon University of Applied Science, Berlin, Germany; Department of Health and Education, Alice Salomon University of Applied Science, Berlin, Germany; Faculty of Health (Department of Human Medicine), Witten/Herdecke University, Witten, Germany; Faculty of Health (Department of Human Medicine), Witten/Herdecke University, Witten, Germany; Faculty of Public Health, Epidemiology and International Public Health, Bielefeld University, Bielefeld, Germany; Faculty of Health (Department of Human Medicine), Witten/Herdecke University, Witten, Germany; Department of Health and Education, Alice Salomon University of Applied Science, Berlin, Germany

**Keywords:** Informal caregiving, Research ethics, Sampling methods

## Abstract

Family caregivers of migrants with dementia constitute a population group that is hard to reach for research participation due to factors such as shame about the disease and past experiences of discrimination. In this article, research-ethical challenges associated with participant recruitment and qualitative data collection among relatives of migrants with dementia are discussed. Over a period of 8 years, 3 studies were conducted to investigate the experiences of family caregivers for persons with dementia of Turkish descent in Germany. Across these studies, a total of 32 family caregivers were interviewed. In this article, based on the “Principles of Biomedical Ethics” according to Beauchamp and Childress (2009), research-ethical conflicts associated with sampling methods and the presence of third parties during qualitative interviews are discussed. The potential risks emanating from sampling strategies and the presence of third parties during interviews regarding the voluntary nature of study participation are examined. Additionally, this article formulates recommendations for ensuring truly voluntary participation and protecting both the participants (family caregivers) and relatives with dementia from harm. These practical recommendations aim to help future researchers to avoid ethical pitfalls and represent a roadmap for making necessary methodological decisions.

An increased awareness of migration-related topics has emerged in medical ethics discourses in recent years, for instance in relation to palliative care (Ilkilic, 2016) or culturally sensitive care (Agbih, 2014). To a lesser extent, ethics-focused research has also begun to take into consideration family caregivers of people with dementia with a migration background (Alzheimer Europe, 2018; Tezcan-Güntekin, 2018a). However, ethical challenges specifically related to the practice of conducting research with hard-to-reach groups have predominantly been examined in relation to nonmigrant target populations (Locher et al., 2006; Phipps, 2002; Sims, 2020) or indigenous people living in Australia (National Health and Medical Research Council, 2018). To the best of our knowledge, there are no previous studies on ethical conflicts regarding the involvement of family caregivers of individuals with migrant backgrounds who have dementia in empirical studies. Therefore, this article will explore research-ethical challenges arising (a) during participant recruitment and (b) during the process of conducting interviews with family caregivers of people with dementia in the presence of other family members or friends. Additionally, recommendations for overcoming these challenges are given.

## Principle-Based Ethics According to Beauchamp and Childress

In the past decades, a principle-based perspective has emerged as the dominant framework within medical ethics. Beauchamp and Childress (2009) developed a principle-based medical ethics perspective based on four principles relevant both to research participation and patient care. The principle-based approach to research ethics implies a process of negotiation between the immediate and overarching benefits of research and possible harm to participants. Additionally, it implies negotiation among the four underlying principles in different contexts. The four principles are (a) autonomy, (b) nonmaleficence, (c) beneficence, and (d) justice. The principle of autonomy can relate to actions taken by a participant or patient, to consent in the context of health care, and participation in research. In order to preserve participant autonomy, researchers should be aware of two aspects throughout the research process. First, participants must be enabled to make decisions regarding their participation free from the influence of other individuals or institutions. Second, patients must be empowered so that they can make autonomous decisions (Beauchamp & Childress, 2009).

Researchers can implement these steps by making information accessible, by ascertaining that participants understand the information provided to them, and by ensuring that participants provide consent voluntarily. Consent must never be obtained by means of coercion.

The principle of nonmaleficence refers to the exclusion of harm during patient care or research participation. Conversely, the principle of beneficence refers to patient or participant well-being. Nonmaleficence and beneficence can stand at odds with each other in the field of medical ethics, for instance when the benefit for patients of a research study is weighed against potential harm for study participants.

The principle of justice refers to research and patient care in the context of social inequality. When ensuring the principle of justice is taken into consideration, researchers might ask whether including a particular group in a research project might lead to disadvantages for another group or whether and to what extent excluding particular groups from research participation due to suspected vulnerability might have negative consequences for visibility and representation.

These four principles are intended to be negotiated so that research designs combine the most important aspects of each of them. This article focuses predominantly on the principles of autonomy, nonmaleficence, and justice. This principle-based ethical framework will structure the following analysis.

## Research Participation of Family Caregivers With Migration Backgrounds

People with a migration background represent a hard-to-reach group for study participation compared to the general population (Lechner et al., 2017; Möhrle et al., 2016; Santos-Hövener et al., 2019). The combination of many factors such as language, education, cultural identification, and fear of institutions can result in barriers to contacting potential participants and requesting their participation (Ibrahim & Sidani, 2014; Saß et al., 2015). Study content and purpose are not always understood, so willingness to participate may be low and even if people are willing to participate, it can be challenging to ensure that the conditions for informed consent are fully met. Disinterest in participating in empirical studies and fears of state surveillance are also factors hindering accessibility, for instance, participants with a migration background may fear that researchers will pass on information about their lifestyles to state institutions in a way that jeopardizes their residency status or health insurance status (Ibrahim & Sidani, 2014). Additionally, family caregivers can be older adult migrants themselves, for instance, in the case of spousal caregiving arrangements (Infratest, 2011; Ulusoy & Graessel, 2017). This group of caregivers is particularly challenging to recruit for study participation, because older age is known to increase difficulties in access to potential participants with migration backgrounds (Kurth & Razum, 2019).

Caregivers face a variety of challenges, including language barriers, fears of discrimination within the health care system, and a lack of knowledge regarding formal support structures (Becker & Mayer, 2011; Kohls, 2012; Tezcan-Güntekin & Razum, 2018). As well as preventing the utilization of formal inpatient or outpatient care services, these factors, in combination with feelings of shame and a high degree of familial responsibility, might function as a barrier to participation in research studies among family caregivers of migrants with dementia (Yilmaz et al., 2009). In this context, use of target-group-oriented strategies, like focused trust and relationship building over time, communication in the native language of the target group, and the participation of individuals who play an important role in the same community, are necessary for successfully including people with migration backgrounds in empirical studies, and it seems to be a good way to do justice to the principle of equity in access to participation (Saß et al., 2015; Yilmaz et al., 2009).

## Conducting Qualitative Interviews in Migrant Communities

Conducting interviews with family caregivers of persons with dementia with migration backgrounds, who can be older migrants themselves, is associated with several research-ethical challenges. For instance, a high degree of reflection that goes beyond everyday experiences is not always common in the older generation of migrants (Tezcan-Güntekin, 2018b). This can lead to potential participants being overwhelmed by the request for an interview, or if they agree to participate, in response to certain questions during the interview. Furthermore, a lack of trust toward researchers may be pronounced among the older migrant population. Skepticism and mistrust must first be reduced through openness and transparency, for instance, by making the research purpose and methods accessible both orally and in simple language or in the native languages of the participants (Tezcan-Güntekin & Özer-Erdogdu, 2021). The aim of transparency is based on the ethical principle of autonomy (Beauchamp & Childress, 2009). Potential participants should be actively empowered to make autonomous decisions about their participation. Autonomous decision making means empowering participants, which can be conducive to building trust. To facilitate autonomous decision making, empowerment, and trust building, interviewers should ideally speak the language of their participants at a native level and should be familiar with participants’ culture. If this is not possible, specially trained language and cultural mediators can be used.

A further challenge associated with interviewing family caregivers of persons with dementia is the presence of the person with dementia or other family members during data collection. The presence of family members or friends has the potential to influence participant responses during an interview situation. Depending on factors such as the nature of the relationship between the participant and the person with dementia could influence how forthcoming a participant might be with sensitive information (Mneimneh et al., 2015). Depending on the cultural context, the likelihood of care being provided at home by relatives may vary; for example, in Germany, 98% of people in need of care who are of Turkish origin are cared for at home by relatives (Okken et al., 2008). This increases the likelihood that the person in need of care will also be present in the home during the interview with the family caregiver and can affect the types of responses (Mneimneh et al., 2018).

## Overview of the Projects and Their Methods

This article reflects on three German research projects involving families of Turkish descent. The purpose of the projects was to analyze the burden, resources, self-management competencies, and support strategies (e.g., self-help and outpatient counseling) experienced and implemented by family caregivers for persons with dementia with migration backgrounds. For Projects 2 and 3, ethical approval was granted by the ethics committees of universities, whereas for Project 1, ethical approval was granted by the ethics committee of an external medical faculty. Thirty-two family caregivers (between the ages of 23 and 78) for older migrants with dementia were interviewed across the three studies; 10 of the caregivers were older adults themselves (nine partners and one mother of a person with dementia). The theoretical concept of self-management posits that chronically ill people can be empowered to manage their own lives despite or with the disease (Haslbeck & Schaeffer, 2007). This approach influenced the design of the interview guides and the data analysis for all projects. Table 1 provides an overview of the studies.

**Table 1.** Overview of the Projects and Key Methodological Aspects

Title of the study	Duration	Study type	Sample	Methods of data collection and analysis
Project 1: Strengthening the self-management competencies of Turkish family caregivers of people with dementia (Bielefeld University)	2013–2016	Research study focusing on family caregivers for people of Turkish descent	12 participants (eight adult children, two wives, one daughter-in-law, one grandchild)	Resources- and problem-centered interviews (Witzel, 2000) and structuring qualitative content analysis (Mayring, 2015)
Project 2: Self-help active—(Inter-)Active self-help for Turkish family caregivers of people with dementia (Alice Salomon University of Applied Science Berlin)	2017–2019	Intervention study	10 participants (seven adult children, two wives, one grandchild)	Problem-centered interviews (Witzel, 2000) and structuring qualitative content analysis (Mayring, 2015)
Project 3: Self-management of caregiving relatives of Turkish origin (Bielefeld University)	2018–2021	Intervention study	11 participants (one older adult mother, four children, two wives, three husbands, one daughter-in-law)	Problem-centered interviews (Witzel, 2000) and structuring qualitative content analysis (Mayring, 2015)

## Ethical and Methodological Challenges Associated With Sampling

### Sampling Methods Used

Four different sampling methods were used across the three studies to reach family caregivers of migrants of Turkish descent with dementia. In the first and second projects, leaflets and letters were used to encourage sampling by “secondary selection,” meaning that persons were provided with the necessary study information and contact details to effectively select themselves, without the researcher choosing exactly who should be taking part (Reinders, 2012, p. 119). Additionally, all three projects used the snowball method of sampling. In the snowball method, the statements of the person initially interviewed are used to develop sampling criteria for further persons to be interviewed (Baur, 2019), or interviewees are asked to recommend further potential participants. The number of participants per snowball was limited to overcome potential bias. Furthermore, sampling by means of gatekeepers like social workers, personnel in mosques, physicians, and nursing care professionals, all with a Turkish migration background, was implemented in all three projects. Interview participants can be reached by so-called “gatekeepers” (Baur, 2019, p. 950), who are important members of a specific community or institution with whom contact already exists or with whom contact seems possible. The gatekeepers forwarded the call for participation to the community, helped researchers to attend possible meeting places such as events in the community, or found participants themselves. Finally, the third project also employed an ethnographic approach, and one of the researchers spend much time while visiting the communities targeted for recruitment.

### Negotiating Insider and Outsider Dynamics

In the first project, the recruitment of interview participants proved to be a challenge. It was easy to establish initial contact with the target group because the researcher speaks Turkish and is perceived to a certain extent as an “insider” (Ergun & Erdemir, 2010). However, the assumption that the cultural background of the researcher (the first author, who is also of Turkish origin) would provide an entry point for field access was called into question. While there was no language barrier, consent to conduct the interview was not automatically given, and the ostensibly shared cultural background was not always sufficient to establish rapport in the presence of other barriers. For instance, the topic of dementia was very shameful for the relatives and they were afraid that talking about it could be understood as complaints about their ill family members. Even after participating in several conversations with the researcher, some of the caregivers made use of their right to refuse to participate in the interview, partly on the grounds that the topic was too personal or that they did not perceive themselves as primary caregivers. An additional barrier was identified in relation to the researcher’s affiliation with a university, which resulted in a sense of distance between her and the participants, possibly because participants interpreted this affiliation as a rejection of the shared community. She was regarded, in this sense, as an outsider of the community; Sims also described this as the concept of “in-group/out-group” (Sims, 2020, p. 693).

### Gatekeeper Sampling

One of the ways these difficulties were dealt with in the first project was by involving intermediaries who acted as gatekeepers. These were two experts, a psychiatrist and the head of an outpatient nursing service, who asked their patients or clients whether they were interested in participating. Gatekeeper sampling was also used in the third project. A partial participatory approach (working together with Turkish nursing care and social work professionals) helped the researchers to find participants for the interviews and intervention. A particularly pivotal role was played by a social worker involved in a social welfare organization, who contacted family caregivers directly. Because this project consisted of an intervention study involving outreach work by health care and nursing professionals, the decision was made to involve professionals throughout various levels of the study design, including recruitment, in order to include their perspectives. While gatekeeper sampling allowed for efficient trust building with participants due to the involvement of community members, it also produced an ethical conflict: Participants may have only agreed to take part in the interviews because they wanted to do the mediating person a favor or because they felt obligated due to being in a relationship of dependence with the gatekeeper. During the first project, a solution to this problem was found by presenting and discussing this conflict with the participants in a session on methodological challenges at a nursing science conference. This peer exchange showed that the level of assurance of voluntariness presented to the participants before the interviews largely compensated for this risk. Additionally, the researcher explicitly assured interviewees in both projects that gatekeepers would in no way be informed about the decision for or against participation and that refusal to participate would not lead to any negative consequences. Nevertheless, the question remains open as to whether the participants took part in the studies due to a sense of obligation.

### Ethnographic Sampling Approach

In the second project, the recruitment process proved to be somewhat less of a barrier because the researcher chose an ethnographic approach at the beginning of the process and spent a lot of time visiting the respective communities. In concrete terms, this meant that she frequently visited groups, associations, and communities and participated in their publicly accessible activities. For example, she visited various religious and cultural institutions such as mosques, Alevi cultural associations, or parents’ associations (Bielefeld Veliler Dernegi) on a monthly or weekly basis. She took part in various programs in the areas of culture, religion, and art (e.g., a class to learn the Ebru art of painting). For ethical reasons, she made her role as a researcher in this process known immediately after making contact. Here it proved to be helpful to emphasize so-called homogeneous characteristics more strongly, for example, to use a dialect that both the interviewer and the interviewee speak in the conversation but also to share knowledge and practices regarding everyday cultural life. This led to the participants gaining confidence in the interviewer and opening up to her. This basis of trust was a prerequisite for participation in an interview, especially due to the sensitivity of the research topic of family caregiving. Through the procedure adopted in the second project, it became clear that a more ethnographically oriented approach to trust building in the initiation of interviews could be a promising way to obtain field access, even with a hard-to-reach target group. Nevertheless, research-ethical principles, especially justice as outlined by Beauchamp and Childress (2009), also play a role in this context. The selection of communities to be visited, and hence given a voice in the study, was carried out by the researchers in advance without consulting with the target group. Additionally, the principle of participant autonomy (Beauchamp & Childress, 2009) can be called into question. While the researcher transparently communicated her role and her intentions in participating in community activities, community members may have come to like the researcher as a person and thus may have felt a sense of obligation to help her by participating in her study. It can be challenging for a researcher in this context to maintain the boundary between their role as a professional and their personal involvement in community activities. For instance, participants might become upset if the researcher ceases to participate in shared activities as soon as they have finished collecting data because they feel they have established a personal connection with the researcher.

## The Presence of Other Family Members and Friends During Interviews

In the context of research involving family caregivers of persons with dementia, the presence of other family members or friends raises ethical concerns on two levels. First, researchers need to consider the potential for harm when persons with dementia are present during the interview and overhear sensitive information about themselves, which can violate the principle of nonmaleficence as outlined by Beauchamp and Childress (2009). Second, situations may arise in which friends or family members other than the person with dementia are present during the interview and may prevent or encourage the sharing of certain information, thus potentially silencing the participant or pressuring them to reveal more than they are comfortable with. This can jeopardize the principle of autonomy (Beauchamp & Childress, 2009), because participants might be pressured to behave in ways that are not consistent with their own preferences.

### Presence of Persons With Dementia

In the first project, the interviewer was faced with an ethical conflict due to the presence of the person with dementia during parts of an interview. At the beginning of the interview, the daughter and the son with whom the interview was conducted were in a separate room, but during the course of the interview, their mother with severe dementia entered. The interview was initially interrupted and then resumed. When the interview was resumed, the mother was still walking around the apartment and occasionally entered the room in which the interview was taking place. The interviewer found herself conflicted as to whether the interview should be continued this way or not. The mother was eventually accompanied to a different part of the apartment by one of the siblings so that the interview could be continued in private.

In the third project, a similar situation occurred. In this case, the interview was continued and completed even though the person with dementia was in the room. Subsequently, the team discussed this issue with other researchers. The results of the discussion were unexpectedly controversial because reference was also made to the caregivers’ duty of supervision, which they may not be able to fulfill when they participate in the interview in a separate room (see protocol in Supplementary Material). In order to resolve these issues, the team decided that they would postpone interview appointments when persons with dementia were present in the room and would schedule new appointments—also providing the option of a telephone interview—for a time when the caregivers could be accessed alone. The team also agreed to inform potential participants early on, while obtaining informed consent, that the person with dementia should not be present during the interview.

### Presence of Other Family Members

In the third project, the team was confronted with another ethical challenge, this time associated with the presence of other family members during the interview, although individual interviews were planned. During one of the interviews, the family caregiver, an older woman caring for his wife, was accompanied by his adult daughters. When the interviewer asked a question about utilizing support structures (self-help groups), the daughters responded negatively and emphasized that utilizing these services did not reflect the wishes of the person with dementia (their father). During the analysis of the interview data, it became apparent that the wife, as the primary caregiver for the person with dementia, was under significant strain and might have accepted external support, had the adult daughters not explicitly dismissed that course of action.

Interestingly, the presence of family members other than the primary caregiver created a group interview situation, which revealed how the dynamics within the family affected the willingness to utilize professional nursing services. While the presence of third parties in this interview situation raises ethical concerns with regard to the participant potentially feeling uncomfortable expressing themself, it also highlights techniques such as group interviewing or joint interviewing as possible avenues for future research in this area.

## Discussion and Conclusion

The insights from the projects have shown that both sampling and the presence of other family members or friends during the data collection process can pose research-ethical challenges. Drawing on the experiences presented above, this section will discuss potential solutions to the ethical challenges raised and formulate recommendations for ethically justifiable conduct in future research practice with family caregivers of migrants with dementia, who may be older migrants themselves.

### Ethical Sampling Approaches: Foregrounding Transparency and Trust Building

The experience made in the first study, belonging to a particular cultural community does not translate into immediate access to this community for research activities, shows an important problem that can arise in a variety of research areas, not only with regard to family caregivers of migrants with dementia. Trust needs to be built actively, for instance, by means of involvement in community activities, as Bonevski (2014) has pointed out in a systematic review. Nevertheless, this process can be facilitated by a certain degree of insider status: “For the insider, shared citizenship, ethnic, linguistic, religious, gender, and cultural identities or simply affinities facilitate the researcher’s access to the field” (Ergun & Erdemir, 2010, p. 18). While researchers can leverage shared diversity characteristics in the process of building trust and rapport with potential participants, the roles of “insider” and “outsider” exist on a spectrum and require constant negotiation in interactions with participants (Ergun & Erdemir, 2010; Sims, 2020). While factors such as shared languages or regions of origin can facilitate field access, this does not mean that researchers who do not have similar diversity characteristics will automatically be denied access (Tezcan-Güntekin & Özer-Erdogdu, 2021). Conversely, advantages due to being an insider in one domain may be counteracted by outsider status in another domain, as described in relation to one of the authors being perceived as an outsider due to her employment at a university.

Thus, sharing aspects of “culture” such as language and place of origin does not equate to understanding a person—“culture” is not a static set of characteristics, but is rather produced, challenged, and reproduced in interactions shaped by intersecting power systems (Knipper, 2014). Having similar experiences or acquiring knowledge about a particular culture are not sufficient by themselves for cultivating relationships with participants based on trust and mutual respect. Rather, competencies relating to self-reflection and communication are necessary to facilitate diversity-sensitive research practices (Grützmann, 2014).

Cooperation between researchers and trusted gatekeepers increases the chance of study participation among hard-to-reach populations and thus constitutes an important methodological tool in ensuring the representation of marginalized groups in research (Saß et al., 2015; Yilmaz et al., 2009). However, it should not be forgotten that access to participants by gatekeepers can also lead to the gatekeeper making a preselection, for example, denying people with low incomes the time and interest to participate and not even asking them to participate (Bonevski et al., 2014), which is an ethical question of justice. Concerning autonomy as a research-ethical principle, conflicts associated with the participants’ sense of obligation toward the gatekeeper and the voluntary nature of their participation can be ameliorated by explicitly referring to the voluntary nature of study participation and pointing out that participation or nonparticipation would in no way be communicated to the expert acting as a gatekeeper. These explanations should be carried out in the native languages of participants and/or in simple language to guarantee that all interviewees, including older adult migrants, can understand. Sims (2020) called this the “ability to participate in the linguistic community of the researched” (p. 699). However, the ethical conflict remains: It cannot be guaranteed that participants did not act out of an implicit sense of obligation when consenting to take part in the study. In this context, it is important to describe and reflect on the problem transparently when presenting the research design to an ethics committee and to point out how exactly gatekeepers are used to ensure that participation is voluntary.

Researchers are obligated to adhere to the principle of autonomous and informed consent in the most exacting manner. This can lead to researchers deciding to exclude people with a low level of education or a lack of basic understanding of what research is in order not to exploit their limited ability to give informed consent. This in turn contradicts the principle of justice because it reifies existing inequalities by elevating the testimony of those who are already privileged by their access to cultural capital. In order to strike a balance between the principles of justice and autonomy, researchers can look toward building more sustainable, empowering relationships with the communities they study; for instance, by taking a more ethnographic approach and “living” with potential participants on their own terms.

Recruiting people with a migrant background by attending routine and well-received leisure activities (such as monthly breakfasts in club communities or regular charity events) is a recognized strategy for attracting participants (Rugkåsa & Canvin, 2011; Tezcan-Güntekin & Özer-Erdogdu, 2021). In the context of Turkish migrant communities in Germany, these types of community-based strategies yielded samples that are diverse in terms of socioeconomic status, educational attainment, and cultural identification (Brand et al., 2019). As an alternative to gatekeeper sampling, the participation of researchers in social activities within the lifeworld of potential interview participants creates trust, which may encourage participation in the study (Tezcan-Güntekin & Özer-Erdogdu, 2021). This, however, requires a generous time allocation for recruiting participants, which must be considered and planned already when applying for research funding. Additionally, this way of sampling can introduce ethical challenges regarding the role of the researcher, who may transparently communicate their professional motives for joining community activities, but nevertheless becomes part of the field as an individual. To prevent feelings of mistrust and betrayal, it is advisable for the researcher not to simply leave the field after data collection, but rather to remain in the community as a contact person, thus consolidating trust not only before, but also after study involvement. Rugkåsa and Canvin (2011) have already identified this challenge for research with ethnic minorities as “reciprocal relationship” (p. 138).

### Ensuring Autonomy and Nonmaleficence Through Preparation and Communication

In relation to the challenge of other family members and friends being present during interviews with family caregivers, a very careful approach is necessary—in communities with a high collective orientation, the notion that a stranger (in this case, the interviewer) would have a say in deciding who should be allowed to be present in a particular space may not be accepted (Mneimneh et al., 2018; Zhou & Nunes, 2013). Additionally, researchers working with family caregivers need to be aware that it may not be comprehensible to the person interviewed why their relative with dementia should not be there. Given that they spend their daily lives in close contact with the person with dementia, often performing tasks related to physical maintenance and hygiene, for example, incontinence or feelings of discomfort when caring for someone, caregivers might not be aware that discussing personal topics, such as incontinence, can make their relative uncomfortable. However, definitively ruling out the presence of the person with dementia during an interview with their family caregiver is associated with additional ethical challenges. The principle of autonomy can be understood to be undermined by taking the right to decide about the presence of their ill relative from the family caregiver. Nevertheless, in the three projects presented here, the researchers leaned toward the exclusion of the person with dementia from the interview situation due to the significant risk of harm; the principle of nonmaleficence was given precedence over the principle of autonomy in this case.

Because the family caregivers invest time and effort in scheduling the interview, a decision on behalf of the researcher to stop and reschedule can threaten the working relationship between the researcher and the interview participant, so a particularly careful procedure is necessary. The researcher can ask in this situation whether the interview could possibly be conducted in another room so as not to disturb or embarrass the person with dementia. If this is refused, questions from the interview that would most likely not bother the ill person can be asked and a request can be made to arrange another (telephone) appointment to discuss the possibly embarrassing or inappropriate questions. Another alternative is to plan the interviews in a neutral place, for example, at a counseling center or in a park. In this case, the researcher can explicitly ask whether the interview participant is intending to bring the person with dementia and if this is the case, assistance by a health or social care professional in another room can be planned. The cost of this type of assistance should already be considered in the financial plan when applying for project funding. The cost of supervision for the research team can be included at this early stage as well, so that the team is better equipped to work on ethical conflicts and resulting stressors.

Additionally, when planning an appointment for a qualitative interview, researchers should emphasize that the interview is meant to be conducted one-on-one, not in the presence of friends or family members, in a private room. This information can be included in participant information documents and consent forms. Researchers might take for granted that participants will come to the interview location alone or arrange for family members (other than the person with dementia) not to enter the room in the home where the interview is taking place. However, researchers should address these requirements explicitly, because participants might have a different understanding of privacy and confidentiality.

Overall, a balance must be found between what is feasible, to ensure that the study can be carried out, and what is justifiable in line with the principles of Beauchamp and Childress (2009), because conducting research-ethically unacceptable research should be out of the question. A transparent and critical approach to ethical challenges, a culture of communication and exchange in research teams, and communication with other experts in the field provide a foundation for developing a constructive and ethically justifiable research strategy in investigations focusing on family caregivers for persons with dementia with migration backgrounds.

## Supplementary Material

gnab179_suppl_Supplementary_Material
