# Supplementary material for: Ethical and Methodological Challenges in Research With Hard-to-Reach Groups: Examples From Research on Family Caregivers for Migrant Older Adults Living With Dementia
Source: Gerontologist. 2021 Dec 7;62(6):823–31. doi: 10.1093/geront/gnab179 (PMC9290906; doi:10.1093/geront/gnab179)
Supplement: gnab179_suppl_Supplementary_Material [file gnab179_suppl_supplementary_material.docx]

# Online Supplementary Material

# Memo/protocol from the team meeting about ethical issues related to the presence of third parties during interviews

Interim evaluation of interviewing practice – discussion with the methods supervision team

Important notes on interview atmosphere to be implemented from the next interview onwards:

The receiver of care (person with dementia) must never, under any circumstances, be present during the interview (same room or in earshot)

If no other arrangement is possible, if they agree, XY (social worker) can supervise the care receiver for the duration of the interview

The caregiver should be alone in the room with the interviewer. Other family members should not be present.

All three recommendations are based on ethical concerns. On the one hand, we cannot be certain how much of the conversation will be understood by the person with dementia and it would simply be immoral to discuss their care while they are present. On the other hand, the interview partner might be inhibited with regards to talking about difficult subject matters.

The recommendation for the interview partner to be alone in a room with the interviewer is also based on ethical aspects. On the one hand, it is good for us when other family members encourage the interview partner to tell us more, however we cannot guarantee that the additional information elicited this way gas truly given voluntarily. The ethics form for the project specified that participants are free to choose what they do and do not want to talk about. If other interview partners are present (for instance other family members), we cannot guarantee this. (Memo/protocol 16.05.2019)
